# Supplementary material for: Dynalign II: common secondary structure prediction for RNA homologs with domain insertions
Source: Nucleic Acids Res. 2014 Nov 21;42(22):13939–48. doi: 10.1093/nar/gku1172 (PMC4267632; doi:10.1093/nar/gku1172)
Supplement: SUPPLEMENTARY DATA [file supp_gku1172_nar-02021-z-2014-File012.zip › manual/Developer/Releases.doc]

**RNAstructure**

**Developer's Manual**

**Building Releases**

**Version 1.0: 09/28/2012**

**Table of Contents**

General Considerations

Building on Unix-based systems

Building on Linux systems

Building on Macintosh systems

Building on Windows systems

**General Considerations**

Building an RNAstructure software release is not a trivial process. Since our focus is reaching as many users as possible, our releases must be optimized for multiple operating systems and software architectures. Multiple builds are necessary because the C++ components of RNAstructure must be compiled into system-specific machine code. The Java components, on the other hand, only need to be built once, and can be built on any desired system, then transplanted into a release bundle at will.

Every RNAstructure release consists of two components for each operating system: a group of text interfaces and a GUI (Graphical User Interface). The text interfaces are built purely in C++, while the GUI has a Java component. A full release for any given operating system consists of two bundles, one that holds the text interfaces and one that holds the GUI.

In addition, the entire RNAstructure repository is released as source code. This bundle is released in two different forms, one for Unix-based systems (Linux and Macintosh) and one for Windows systems.

ALL TARBALLS THAT HAVE EXECUTABLES IN THEM SHOULD BE TESTED BEFORE RELEASE IN ALL POSSIBLE OPERATING SYSTEMS WITH WHICH THEY CAN BE USED.

All building guides assume that the repository is ready to be built as is--that all files are properly updated for release, all manuals are up to date, and all programs run properly. A complete RNAstructure release involves building the following files on the three major operating systems. More will be explained about each of these later:

1. Unix-based systems
   1. Linux
      1. 32-bit
         1. RNAstructureLinuxTextInterfaces.tgz
         2. RNAstructureForLinux.tgz
      2. 64-bit
         1. RNAstructureLinuxTextInterfaces64bit.tgz
         2. RNAstructureForLinux64bit.tgz
   2. Macintosh
      1. RNAstructureTextInterfacesMac.tgz
      2. RNAstructureForMac.tgz
   3. RNAstructureSource.tgz
2. Windows systems
   1. 32-bit
      1. RNAstructureWindowsTextInterfaces.zip
      2. RNAstructure.zip
   2. 64-bit
      1. RNAstructureWindowsTextInterfaces64bit.zip
      2. RNAstructure64bit.zip
   3. RNAstructureSource.zip

**Building on Unix-based systems**

All Unix-based systems have a similar operating system framework, although some differences exist between different distributions and variations of the parent system. Both Linux and Macintosh operating systems are based on Unix principles. So, much of the build process between the two operating systems is similar. However, the final executables needed for Linux and Macintosh differ substantially (more details will be explained about this later).

Releases on Unix-based systems can be built with the Makefiles included in the RNAstructure repository. For the initial build, no third-party software is necessary. Third-party software is only necessary at the final build steps, and only on Macintosh.

The Unix-based builds give the developer a lot of control to specify exactly how releases are compiled and built. In general, it is best to follow the specific build processes defined in this manual, because they have been thoroughly tested and used to build many previous releases of RNAstructure.

The majority of the RNAstructure builds are done on Unix-based systems.

A source tarball, RNAstructureSource.tgz, is also released. For completeness, how to make this tarball is described in both the Linux and Mac build sections. However, this tarball only needs to be made once per RNAstructure release; it can be read on either Linux or Macintosh systems.

**Building on Linux Systems**

Creating an RNAstructure build for Linux systems works exactly the same way whether the current Linux operating system is 32-bit or 64-bit. The following list of steps details how to build a Linux RNAstructure release, starting with the text interfaces and then moving on to the GUI. For the sake of clarity, each major section of the build starts with its own fresh checkout of RNAstructure from CVS. While that is not required, it is a good idea to do for the sake of organization during the build. Making sure that each step has its own sandbox also ensures that the right files are included in each particular build executable.

Building the text interfaces

1. Create a directory called “linuxText” and go into it.
2. Check out a fresh version of the RNAstructure repository using cvs and go into the RNAstructure folder, by executing this command: cvs co RNAstructure; cd RNAstructure.
3. Type make all. This will build all the text interfaces into the RNAstructure/exe directory. This includes all serial and SMP interfaces.
4. Type make clean. This will delete all object files and temporary files that may have been made during the build.
5. Execute the following exactly as written to remove all CVS directories from the RNAstructure folder and its subfolders: find . -depth -name CVS | xargs rm -rf
6. Move up one directory to the linuxText folder.
7. Execute the following command to compress the RNAstructure folder into a gzipped tarball: tar -zvcf LinuxBuild.tgz RNAstructure
8. If building on a 32-bit system, rename the tarball to RNAstructureLinuxTextInterfaces.tgz. If building on a 64-bit system, rename the tarball to RNAstructureLinuxTextInterfaces64bit.tgz.
9. Move the tarball out of the “linuxText” directory to wherever it needs to be.
10. Delete the “linuxText” directory, since it's not necessary anymore.

Building the GUI

1. Create a directory called “linuxGui” and go into it.
2. Create a directory called “tempGui” and go into it.
3. Check out a fresh version of the RNAstructure repository and go into the RNAstructure folder, by executing this command: cvs co RNAstructure; cd RNAstructure..
4. In compiler.h, set the Java include paths. The file has instructions inside for how to do this.
5. Go into the RNAstructure_java_interface directory.
6. Type make GUI. This will build the Java GUI into the RNAstructure/exe directory.
7. Delete RNAstructure.bat from the RNAstructure/exe directory.
8. Copy the following files and folders from the RNAstructure directory to the tempGui directory: data_tables, examples, exe, manual/GUI (only copy the GUI folder, not the manual folder as well), Version.txt
9. Move up one directory to the tempGui folder.
10. Delete the RNAstructure folder.
11. Rename the GUI folder to manual.
12. Move up one directory to the linuxGui folder.
13. Rename the tempGUI folder to RNAstructure.
14. Execute the following command to compress the RNAstructure folder into a gzipped tarball: tar -zvcf LinuxBuild.tgz RNAstructure
15. If building on a 32-bit system, rename the tarball to RNAstructureForLinux.tgz. If building on a 64-bit system, rename the tarball to RNAstructureForLinux64bit.tgz.
16. Move the tarball out of the “linuxGui” directory to wherever it needs to be.
17. Delete the “linuxGui” directory, since it's not necessary anymore.

Creating the source code bundle

1. Create a directory called “linuxSource” and go into it.
2. Check out a fresh version of the RNAstructure repository and go into the RNAstructure folder.
3. Execute the following exactly as written to remove all CVS directories from the RNAstructure folder and its subfolders: find . -depth -name CVS | xargs rm -rf
4. Move up one directory to the linuxSource folder.
5. Execute the following command to compress the RNAstructure folder into a gzipped tarball: tar -zvcf RNAstructureSource.tgz RNAstructure
6. This only needs to be done once, on either a 32-bit or 64-bit Linux system, but not both.
7. Move the tarball out of the “linuxSource” directory to wherever it needs to be.
8. Delete the “linuxSource” directory, since it's not necessary anymore.

Overall process

To create a full Linux release, the following things should be done:

1. Build a text interface tarball (as above) on a 32-bit system
2. Build a Java GUI tarball (as above) on a 32-bit system
3. Build a text interface tarball (as above) on a 64-bit system
4. Build a Java GUI tarball (as above) on a 64-bit system
5. Build a repository source tarball (as above) on either a 32 or 64 bit system. It doesn't matter which, just use whichever is most convenient.

The following files should have been created:

1. RNAstructureLinuxTextInterfaces.tgz (32-bit text interfaces)
2. RNAstructureForLinux.tgz (32-bit Java GUI)
3. RNAstructureLinuxTextInterfaces64bit.tgz (64-bit text interfaces)
4. RNAstructureForLinux64bit.tgz (64-bit Java GUI)
5. RNAstructureSource.tgz (Repository tarball in Unix format)

**Building on Macintosh Systems**

Creating an RNAstructure build for Macintosh systems is done similarly to Linux systems when text interfaces are built, but differently when building the GUI. The following list of steps details how to build a Macintosh RNAstructure release, starting with the text interfaces and then moving on to the GUI. For the sake of clarity, each major section of the build starts with its own fresh checkout of RNAstructure from CVS in a fresh directory. While that is not required, it is a good idea to do for the sake of organization during the build. Making sure that each step has its own sandbox also ensures that the right files are included in each particular build executable.

Building the text interfaces

1. Create a directory called “macText” and go into it.
2. Check out a fresh version of the RNAstructure repository and go into the RNAstructure folder.
3. In compiler.h, set the Mac operating system name, compiler flags, and linking flags. The file has instructions inside for how to do this.
4. Type make all. This will build all the text interfaces into the RNAstructure/exe directory.
5. Type make clean. This will delete all object files and temporary files that may have been made during the build.
6. Execute the following exactly as written to remove all CVS directories from the RNAstructure folder and its subfolders: find . -depth -name CVS | xargs rm -rf
7. Move up one directory to the macText folder.
8. Execute the following command to compress the RNAstructure folder into a gzipped tarball: tar -zvcf MacBuild.tgz RNAstructure
9. Rename the tarball to RNAstructureTextInterfacesMac.tgz.
10. Move the tarball out of the “macText” directory to wherever it needs to be.
11. Delete the “macText” directory, since it's not necessary anymore.

Building the GUI

1. Create a directory called “macGui” and go into it.
2. Create a directory called “tempGui” and go into it.
3. Check out a fresh version of the RNAstructure repository and go into the RNAstructure folder.
4. In compiler.h, set the Mac operating system name, compiler flags, and linking flags. The file has instructions inside for how to do this.
5. In compiler.h, set the Java include paths. The file has instructions inside for how to do this.
6. Go into the RNAstructure_java_interface directory.
7. Type make GUI. This will build the Java GUI into the RNAstructure/exe directory.
8. Use the JAR Bundler program, which comes standard on Mac, to create a Mac application bundle that is placed in the tempGUI directory. (/usr.share/java/Tools/Jar Bundler)
   1. Open JAR Bundler.
   2. Click on the “Build Information” tab.
   3. To identify the main class, click the “Choose” button and select RNAstructure.jar from the RNAstructure/exe directory. The JAR Bundler application can pull the main class out of the JAR's manifest file (which is automatically built into it).
   4. Select “Use Macintosh Menu Bar”, “Anti-alias Text”, and “Anti-alias Graphics”.
   5. Set the JVM version as “1.6+” (Java 1.6 or later).
   6. Set the bundle icon by selecting “Choose Icon” and selecting “Icon.icns” from the RNAstructure/RNAstructure_java_interface/images directory.
   7. Click on the “Classpath and Files” tab.
   8. Under “Additional Files and Resources”, use the “Add” button to select:
      1. All data files from the RNAstructure/data_tables directory
      2. libRNAstructure_GUI.dylib and RNAstructure.jar from the RNAstructure/exe directory
      3. Splash.gif from the RNAstructure_java_interface/images directory
   9. Click on the “Properties” tab.
   10. Set the bundle name as RNAstructure. Specify the version and Get-Info string.
   11. Click to set the working directory inside the package.
   12. Under “Additional Properties”, create a key “SplashFile” with value $JAVAROOT/Splash.gif. Move this from Java Properties to Java, by right-clicking on the final package, and selecting show contents. Then right-click on info.plist and make the change from Java Properties to Java.
   13. Click “Create Application.” In the dialog box that pops up, select tempGui as the folder that will hold the application bundle, and RNAstructure as the file name.
   14. Click “Create”. The dialog box will disappear. Check to make sure the bundle is in the right place; if it is, close JAR bundler.
9. Copy the following files and folders from the RNAstructure directory to the tempGui directory: examples, manual/GUI (only copy the GUI folder, not the manual folder as well), Version.txt, and examples.
10. Move up one directory to the tempGui folder.
11. Delete the RNAstructure folder.
12. Rename the GUI folder to manual.
13. Move up one directory to the macGui folder.
14. Rename the tempGUI folder to RNAstructure.
15. Execute the following command to compress the RNAstructure folder into a gzipped tarball: tar -zvcf RNAstructureForMac.tgz RNAstructure.
16. Move the tarball out of the “macGui” directory to wherever it needs to be.
17. Delete the “macGui” directory, since it's not necessary anymore.

Creating the source code bundle

1. Create a directory called “macSource” and go into it.
2. Check out a fresh version of the RNAstructure repository and go into the RNAstructure folder.
3. Execute the following exactly as written to remove all CVS directories from the RNAstructure folder and its subfolders: find . -depth -name CVS | xargs rm -rf
4. Move up one directory to the macSource folder.
5. Execute the following command to compress the RNAstructure folder into a gzipped tarball: tar -zvcf RNAstructureSource.tgz RNAstructure
6. Move the tarball out of the “macSource” directory to wherever it needs to be.
7. Delete the “macSource” directory, since it's not necessary anymore.

Overall process

To create a full Macintosh release, the following things should be done:

1. Build a text interface tarball (as above)
2. Build a Java GUI tarball (as above)
3. Build a repository source tarball (as above)

The following files should have been created:

1. RNAstructureTextInterfacesMac.tgz (Text interfaces)
2. RNAstructureForMac.tgz (Java GUI)
3. RNAstructureSource.tgz (Repository tarball in Unix format)

**Building on Windows systems**

The Windows native programs are built using Microsoft Visual Studio and the Intel C Compiler. This program builds both 32-bit and 64-bit versions of the text interfaces and a Windows GUI. The source code is also released as a zip file in DOS format.

Note that the use of openmp now requires distribution of libiomp5.dll from \Program Files\Intel\Composer XE\redist\[Intel64 or ia32]\compiler\libiomp5.dll.

With the text interfaces, this dll should be placed in the exe directory with the executables.
